# Neuropsychiatric Presentation in Indirect Carotid-Cavernous Fistula: A Case Report

**DOI:** 10.7759/cureus.37523

**Published:** 2023-04-13

**Authors:** Chong Ying Sze, Salinawati Bakin, Rozita Ismail

**Affiliations:** 1 Ophthalmology, Hospital Serdang, Kajang, MYS; 2 Interventional Radiology, Hospital Kuala Lumpur, Kuala Lumpur, MYS

**Keywords:** digital subtraction angiography(dsa), endovascular embolisation, indirect, neuro-psychiatric, neuro-ophthalmic manifestations, neuro radiology, carotid-cavernous sinus fistula, case report

## Abstract

Carotid-cavernous fistula (CCF) is a rare sight and potentially life-threatening disorder arising from an abnormal connection between the carotid artery and the cavernous sinus. It can be classified into direct or indirect according to different arteriovenous shunts. Direct CCF usually has dramatic ocular presentations, whereas indirect CCF has a more insidious course and may be associated with neurologic symptoms in posteriorly draining fistulas.

A 61-year-old gentleman presented with five days history of altered behavior and double vision preceding a bulging left eye. Ocular examination showed left eye proptosis, generalized chemosis, total ophthalmoplegia, and raised intra-ocular pressure. Computed tomography angiography (CTA) brain and orbit demonstrated dilated superior ophthalmic vein (SOV) with communication to a tortuous cavernous sinus suggestive of carotid-cavernous fistula (CCF). Digital subtraction angiography (DSA) eventually confirmed the presence of indirect communication between branches of the bilateral external carotid artery (ECA) and left cavernous sinus, which is a type C indirect CCF according to the Barrow classification. Total embolization of left CCF was successfully achieved via transvenous access. A marked reduction of proptosis and intra-ocular pressure was noted following the procedure.

Although rare, neuropsychiatric presentation could be a possible presentation of CCF, and treating physicians should be aware of it. A high index of suspicion and prompt diagnosis is crucial in managing this sight and life-threatening condition. Early intervention can improve the prognosis of patients.

## Introduction

CCF is a rare sight and a potentially life-threatening disorder arising from an abnormal connection between the carotid artery and the cavernous sinus [1]. It can be classified based on the hemodynamic properties, etiology, or anatomy of the shunt [2]. Barrow et al. classified CCF into direct fistula (type A) and indirect fistulas (type B, C, D) [3]. Direct CCF usually has dramatic ocular presentations, clinically characterized by Dandy’s triad (pulsatile proptosis, conjunctival chemosis, and ocular bruit) [4]. Indirect CCF, however, has a more insidious onset and may be associated with neurologic symptoms in posteriorly draining fistulas [5]. CCF with neuropsychiatric symptoms is unusual and rarely reported. Here, we would like to report a case of CCF with neuropsychiatric symptoms and discuss its pathophysiology and management.

## Case presentation

A 61-year-old gentleman with a medical history of dyslipidemia presented to the emergency department with double vision for the past month, followed by five days history of altered behavior and bulging left eye. Further history by family members revealed that the patient complained of slurred speech and abnormal behavior of shouting randomly and running around naked at home.

**Figure 1:**
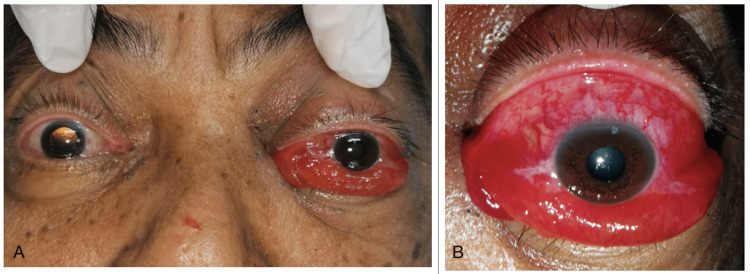
Patient with left eye proptosis, conjunctival injection, and generalised chemosis in left-sided dural CCF (A) and prominent episcleral corkscrew vessels (B). The patient also had total ophthalmoplegia due to ocular motor paresis. CCF = carotid-cavernous fistula

Ocular examination of the left eye (Figure 1A-1B) showed marked proptosis, conjunctival injection, generalized chemosis, prominent episcleral corkscrew vessels on the superior conjunctiva, and total ophthalmoplegia. His relative afferent pupillary defect was positive with high intra-ocular pressure of 50mmHg. He was started on topical anti-glaucomas to manage the raised intra-ocular pressure. Unfortunately, his visual acuity and color vision could not be assessed due to his altered mental status on presentation with a Glasgow Coma Scale (GCS) of 14 (confusion). The rest of his neurological examination was normal. No significant history of head trauma was reported. There were no known underlying connective tissue disorders in the family as well. 

**Figure 2:**
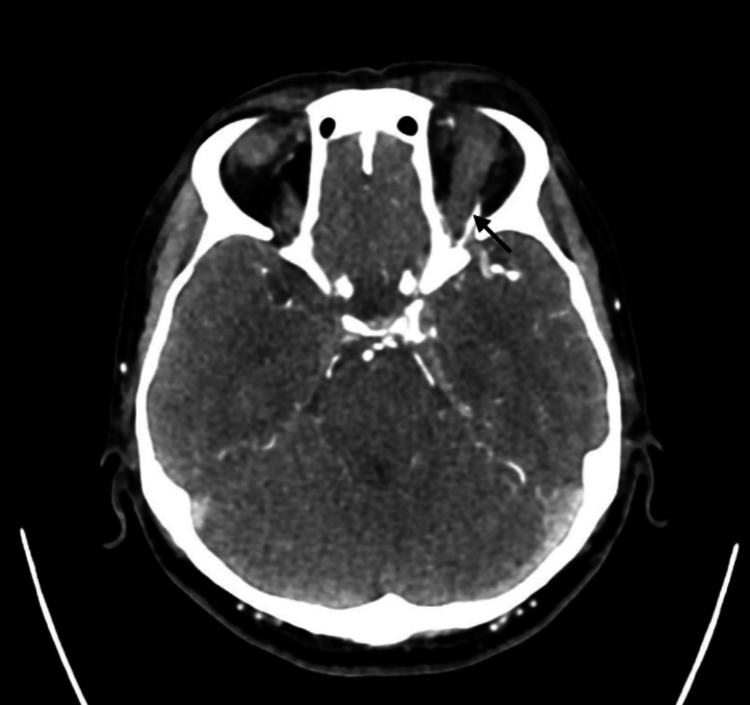
Dilated and tortuous left superior ophthalmic vein (SOV) noted during Computed Tomography Angiography (CTA) of the brain and orbit.

Due to his neuropsychiatric presentation, the medical team initiated intravenous ceftriaxone and acyclovir to cover for possible meningoencephalitis. Negative results were obtained for routine blood tests, erythrocyte sedimentation rate (ESR), C-reactive protein, and blood cultures. A lumbar puncture was performed, and cerebrospinal fluid (CSF) analysis tests revealed normal findings for cell counts, protein, and glucose, as well as negative PCR results for herpes simplex virus (HSV) type 1 and 2. CTA of the brain and orbit (Figure 2) demonstrated a dilated superior ophthalmic vein (SOV) with communication to the cavernous sinus and tortuous cavernous segment of the left internal carotid arteries (ICA), suggesting CCF. On the third day of admission, his GCS dropped further to E1V2M5 in the evening, and he was intubated for airway protection. The neurosurgery team was promptly consulted, and the patient was subsequently transferred to another tertiary center with the expertise of interventional radiology. 

**Figure 3:**
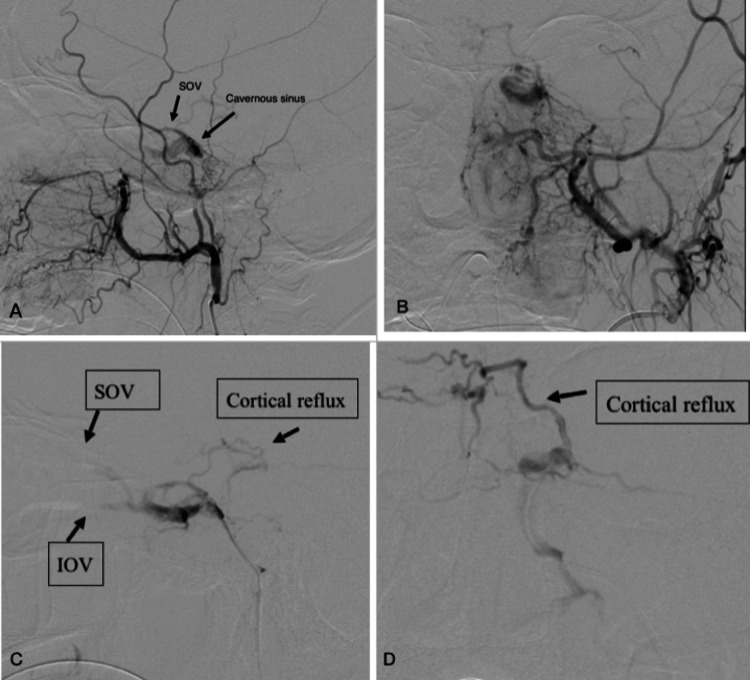
Pre-embolization digital subtraction angiography (DSA) of the left external carotid artery (ECA) showed early filling of the left cavernous sinus on arterial phase in lateral (A) and AP (B) views. Pre-embolization run from the microcatheter following successful cannulation of the inferior petrosal sinus into the posterior part of the cavernous sinus better delineates the cavernous sinus, ophthalmic veins, and cortical reflux in lateral (C) and AP (D) views.

Digital subtraction angiography (DSA), followed by embolization, was performed under general anesthesia. Vascular accesses were gained from the right common femoral artery and vein. Catheterization of all cerebral arteries was performed using a 4Fr JB 2 catheter. Cerebral DSA ultimately showed the presence of indirect communication between bilateral middle meningeal and terminal internal maxillary branches of the bilateral ECA and left cavernous sinus, indicating an indirect type C dural CCF according to the Barrow classification. Abnormal filling of the left cavernous sinus in the arterial phase was noted on bilateral external carotid artery angio-runs (Figure 3A-3B). Due to the indirect nature of the left CCF, endovascular treatment of CCF was done via transvenous access. A JB2 diagnostic catheter was positioned in the left external carotid artery as a trans-arterial navigation in the venous system. Transvenously, a 4Fr Vertebral catheter and 2.7Fr Progreat micro-catheter were used to cannulate the left cavernous sinus via the left inferior petrosal sinus. A pre-embolization run from the micro-catheter following successful cannulation of the inferior petrosal sinus into the posterior part of the cavernous sinus delineated the cavernous sinus, ophthalmic veins, and cortical reflux in lateral (Figure 3C) and AP (Figure 3D) views. 

**Figure 4:**
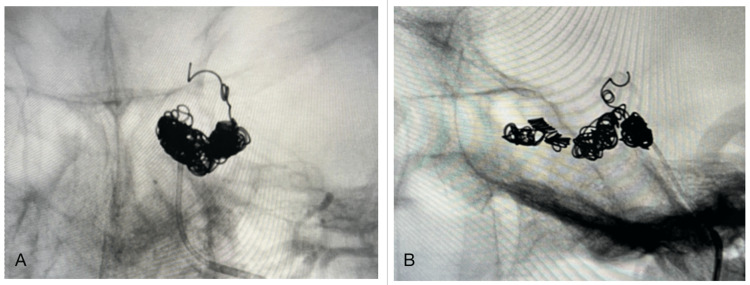
Embolization of the left cavernous sinus using MicroNester Pushable coils, AP (A), and lateral (B) views.

**Figure 5:**
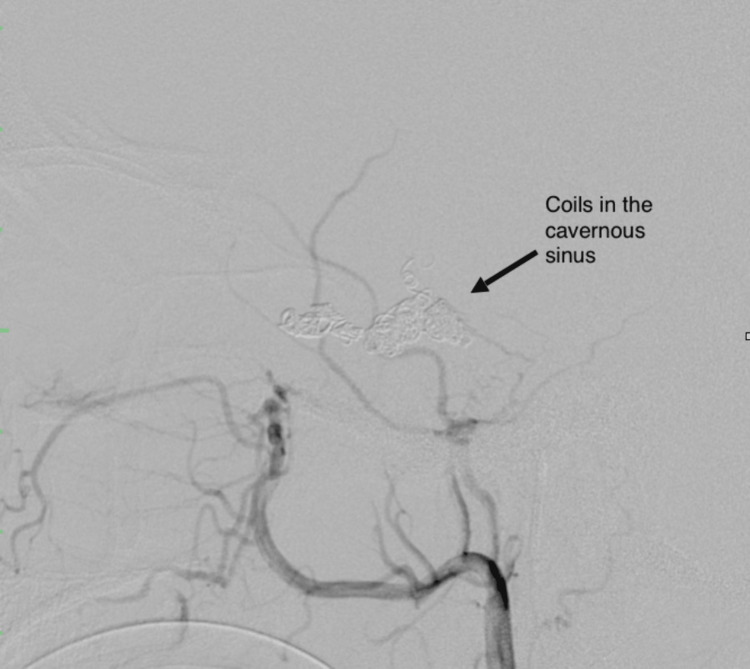
Post-embolisation control run from left ECA in lateral view showed total embolization of the left CCF achieved. ECA = external carotid artery CCF = carotid-cavernous fistula

A total of 9 MicroNester pushable coils were implanted into the posterior part of the left SOV, cavernous sinus, and proximal feeder of the cortical reflux vessel (Figure 4A-4B). On control angiography from the left ECA, total embolization of the left CCF was achieved (Figure 5). 

**Figure 6:**
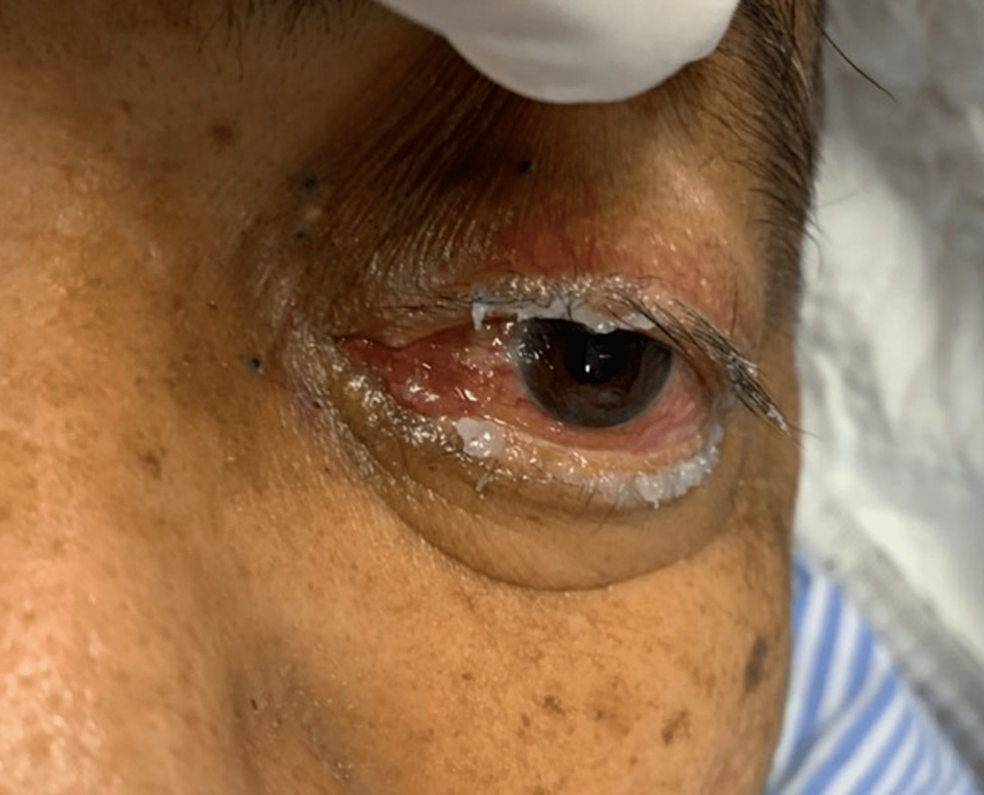
The patient on day one post-embolization showed a marked reduction in left eye proptosis, conjunctival injection, and chemosis.

A follow-up review of the patient on day one post-embolization showed a marked reduction of his left eye proptosis, conjunctival injection, and chemosis (Figure 6). His left eye intra-ocular pressure was controlled with four topical anti-glaucomas and remained stable at 20mmHg during subsequent ophthalmology follow-up. He was extubated on day three following the procedure. Although his cognitive function had improved, neurological examination revealed the patient sustained speech and motor deficits with slurring of speech and difficulty ambulating. 

## Discussion

Clinical findings in CCF depend on the flow type and anatomy of the arteriovenous shunt. Direct CCF is characterized by direct communication between the ICA and the cavernous sinus. They are usually high flow with acute presentation and typically caused by trauma or spontaneous rupture of an ICA aneurysm [5]. Clinical signs in CCFs include pulsating proptosis, red eye with arterialization of the conjunctival and episcleral vessels, chemosis, strabismus due to ocular motor nerve dysfunction, orbital congestion, raised intraocular pressure, and optic neuropathy [5]. Indirect or dural CCFs are caused by communication between the dural branches of the carotid arteries and the cavernous sinus. They are usually low flow and have a slow and progressive course, which matches the clinical history of this patient, who presented with double vision for one month before the diagnosis was made. Neuropsychiatric presentation [6] in CCF, such as this patient's altered behavior and mental status, is rare.

Any cerebral disorder may cause psychiatric symptoms. Neuropsychiatric symptoms could be defined as psychiatric manifestations in cerebral disorders. Characteristics of neuropsychiatric symptoms include anxiety, neurotic complaint, apathy, mood disorder, hallucinations, delusions, behavioral and personality changes, delirium, and cognitive impairment. These symptoms usually occur concurrently in cerebral disorders [6]. 

To understand its pathophysiology, we need to know the venous drainage of the cavernous sinus. CCFs drain toward the anterior via ophthalmic veins, inferior via pterygoid plexus and inferior petrosal sinus (IPS), contralateral via inter-cavernous connections, posterior via the deep venous system, superior petrosal sinus (SPS), and cerebellar veins, and superior via a superficial middle cerebral vein (SMCV). In both direct and indirect CCFs, anterior and inferior drainages were the most common and, thus, usually presented with marked ocular signs. Posterior and superior drainages, however, were noted only in long-standing high-flow direct CCFs [7]. Posteriorly draining fistulas may be associated with neurologic symptoms, e.g., confusion and expression of aphasia in dural CCFs [8-10]. 

This patient's DSA findings (Figure 4) revealed the presence of cortical venous reflux (CVR) with indirect communication between the bilateral middle meningeal and terminal internal maxillary branches of the bilateral ECA in the cavernous sinus. The obliteration or stenosis of venous drainage routes leads to a converging venous outflow that develops into CVR and results in venous congestion of the brain parenchyma [11]. Our patient's cognitive decline can be attributed to venous congestive encephalopathy, a term introduced in 1994 that describes cranial neurologic deficits caused by venous hypertension [12]. 

Due to similar clinical presentation, excluding cavernous sinus thrombosis (CST) as an important differential diagnosis is essential. CST is notorious for its bad prognosis and known to have a mortality rate as high as 30% in the antibiotic era [13]. Systemic workups, including full blood count, electrolytes, coagulation profile, blood cultures, HSV, and CSF analysis, were crucial to rule out possible infective causes, electrolyte imbalances, and thromboembolic conditions from coagulation disorders. Non-invasive imaging, such as computed tomography (CT), magnetic resonance (MR), CTA, and MR angiography (MRA), are helpful in the initial workup of possible CCF. CT scan of the orbit may show proptosis, dilated superior ophthalmic vein (SOV), and enlargement of the ipsilateral cavernous sinus. While CTA can detect the presence of CCF and reveal any filling defect in the cavernous sinus, it cannot provide information on blood-flow characteristics in fistulas [14]. T2-weighted MRI will help delineate hyperintensity areas in the venous territory as direct evidence of venous congestion [8]. DSA remains the gold standard in CCF to localize lesions accurately, classify, and plan for endovascular intervention [14].

Although dural CCFs may have spontaneous resolution without treatment, CCFs associated with CVR are at a higher risk of venous infarction or hemorrhage than those without CVR [9]. Therefore, CVR on DSA indicates urgent endovascular treatment as the natural history of untreated dural CCF with CVR is associated with a poor prognosis with an annual mortality rate of 3.8% [15]. Endovascular treatment with embolization is the most common approach to obliterate the connection in symptomatic and high-flow fistulas.

## Conclusions

Neuropsychiatric presentation in carotid-cavernous fistula is rare and points towards posteriorly draining CCFs. The importance of highlighting this unusual presentation in CCF is so that treating physicians will be more aware and not miss this crucial differential diagnosis other than CST, as both have completely different management with dire sequelae. CCF with cortical venous reflux carries a poor prognosis and thus, should be treated urgently to obliterate the fistula. Prompt diagnosis is crucial to managing this sight-threatening condition and minimizing potentially debilitating neurological sequelae risks. Multi-disciplinary efforts between the ophthalmology, medical, interventional radiology, and neurology team are essential to ensure the best outcome for the patient.

## Appendices

**Table 1:** Classification of carotid-cavernous fistulas (CCFs) according to Barrow et.al.

Type	Flow Types	Site of arteriovenous shunt
A	Direct	Connection between ICA and cavernous sinus
B	Indirect	Connection between dural branches of ICA and cavernous sinus
C	Indirect	Connection between dural branches of ECA and cavernous sinus
D	Indirect	Connection between dural branches of both ICA and ECA and the cavernous sinus
